# Metabolic Modulation of Intracellular Ammonia via Intravesical Instillation of Nanoporter‐Encased Hydrogel Eradicates Bladder Carcinoma

**DOI:** 10.1002/advs.202206893

**Published:** 2023-02-12

**Authors:** Weiqiang Jing, Chen Chen, Ganyu Wang, Maosen Han, Shouzhen Chen, Xin Jiang, Chongdeng Shi, Peng Sun, Zhenmei Yang, Benkang Shi, Xinyi Jiang

**Affiliations:** ^1^ Department of Urology Qilu Hospital Cheeloo College of Medicine NMPA Key Laboratory for Technology Research and Evaluation of Drug Products and Key Laboratory of Chemical Biology (Ministry of Education) Department of Pharmaceutics School of Pharmaceutical Sciences Cheeloo College of Medicine Shandong University Cultural West Road Jinan Shandong Province 250012 China; ^2^ Shandong University of Traditional Chinese Medicine University Road Jinan Shandong Province 250355 China

**Keywords:** bladder carcinoma, carbamoyl phosphate synthetase 1, metabolic modulation, urea, urea transporter‐B

## Abstract

Tumor protein 53 (TP53) mutation in bladder carcinoma (BC), upregulates the transcription of carbamoyl phosphate synthetase 1 (CPS1), to reduce intracellular ammonia toxicity. To leverage ammonia combating BC, here, an intravesically perfusable nanoporter‐encased hydrogel system is reported. A biomimetic fusogenic liposomalized nanoporter (FLNP) that is decorated with urea transporter‐B (UT‐B) is first synthesized with protonated chitosan oligosaccharide for bladder tumor‐targeted co‐delivery of urease and small interfering RNA targeting CPS1 (siCPS1). Mussel‐inspired hydrogel featured with dual functions of bio‐adhesion and injectability is then fabricated as the reservoir for intravesical immobilization of FLNP. It is found that FLNP‐mediated UT‐B immobilization dramatically induces urea transportation into tumor cells, and co‐delivery of urease and siCPS1 significantly boosts ammonia accumulation in tumor inducing cell apoptosis. Treatment with hybrid system exhibits superior anti‐tumor effect in orthotopic bladder tumor mouse model and patient‐derived xenograft model, respectively. Combined with high‐protein diet, the production of urinary urea increases, leading to an augmented intracellular deposition of ammonia in BC cells, and ultimately an enhanced tumor inhibition. Together, the work establishes that cascade modulation of ammonia in tumor cells could induce tumor apoptosis and may be a practical strategy for eradication of TP53‐mutated bladder cancer.

## Graphical abstract

Fusogenic liposomalized nanoporter (FLNP)‐mediated urea transporter‐B immobilization dramatically enhances urea transport into tumor cells, and codelivery of urease and small interfering RNA targeting carbamoyl phosphate synthetase 1 significantly boosts ammonia accumulation in tumor inducing cell apoptosis. A mussel‐inspired hydrogel is fabricated as the reservoir for intravesical immobilization of FLNP.

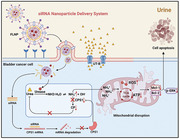

## 1 Introduction

Bladder carcinoma (BC) is the most common neoplasm of the urinary tract, with ≈573 000 new cases and 213 000 deaths per year, globally.^[^ ^1^ ^]^ Non‐muscle‐invasive bladder cancer (NMIBC) accounts for ≈70% of all bladder cancers.^[^ ^2^ ^]^ Despite intensive treatment with intravesical instillation of chemotherapeutics and radical cystectomy following transurethral tumor resection, two‐thirds of patients with NMIBC will suffer recurrence and even disease progression to muscle‐invasive bladder cancer, among which over ≈55% of patients die within 5 years regardless any of additional treatments.^[^ ^3^ ^]^ It is imperative to develop effective strategies for targeting and eliminating the refractory neoplastic burden within the bladder, which remains elusive.

**Figure 1:**
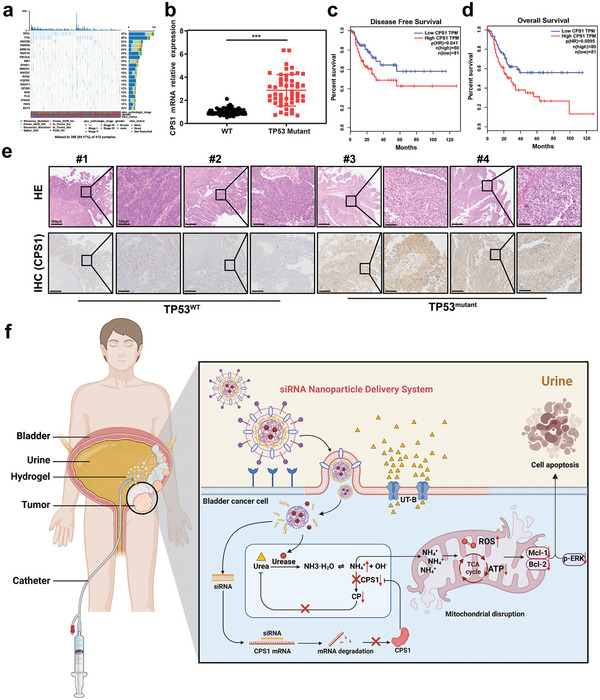
CPS1 expression is almost always increased together with TP53 mutation in bladder cancer. a) Primary genetic alterations in bladder cancer patients. b) Quantification of CPS1 mRNA levels in human TP53 wild‐type (*n* = 50) and TP53‐mutant (*n* = 50) tumor tissue samples. Student's *t*‐test. Curves showing the c) disease‐free survival and d) overall survival of CPS1 high (*n* = 80) and low (*n* = 81) expression bladder cancer patients. The number of n indicates biologically independent samples. e) Two representative HE and IHC images of CPS1 expression in human TP53 wild‐type and TP53‐mutant tumor tissue samples. Scale bars in low magnification images represent 500 µm, and in high magnification images represent 100 µm. f) Schematic illustration of the intravesical instillation of the nanoparticle‐hydrogel system in bladder cancer that initiates the accumulation of ammonium ions in the cells and damage to mitochondria and ultimately leads to the apoptosis of tumor cells. Data are presented as mean ± s.d. ***p < 0.001 by Student's *t*‐test.

Mutation of tumor protein 53 (TP53) is the most frequent genetic alteration in bladder cancer, which results in transcriptional activation, senescence, apoptosis, and changes in metabolism,^[^ ^4^, ^5^ ^]^ promoting tumor progression (**Figure** **1**a). Carbamoyl phosphate synthetase 1 (CPS1), a multidomain mitochondrial rate‐limiting enzyme protein, catalyze the first committed step of the urea recycle by converting ammonia into carbamoyl phosphate for ammonia detoxification and disposal.^[^ ^6^ ^]^ We found that in TP53‐mutant BC, the CPS1 expression level was significantly upregulated (Figure 1b). Moreover, stratification of patients by CPS1 expression revealed a disease free and overall survival advantage for patients with lower CPS1 expression (Figure 1c,d). Consistent with the bioinformatic data, CPS1 protein levels in TP53‐mutant tumor tissues were also considerably elevated compared with those in TP53‐wild‐type tumor tissues in BC patients (Figure 1e). These data indicate that TP53‐mutant bladder cancer considerably upregulates the transcription of CPS1 to promote ammonia metabolism in cancerous cells, which may otherwise be toxic to them. These results have thus inspired us to conclude that harnessing the intracellular accumulation of ammonia might be an amenable approach to eradicate BC.

Bladder tumors usually originate from the urothelium and are directly exposed to concentrated urinary urea,^[^ ^7^ ^]^ which can be catalyzed into cytotoxic ammonia via urease. Orientated polarization of urea toward tumors is first and foremost essential for modulating cytotoxic ammonia for combating BC. Urea transporter‐B (UT‐B), a passive membrane channel, is highly expressed in the membrane of erythrocytes and some epithelial and endothelial cells, for example, 14 000 UT‐B per erythrocyte.^[^ ^8^, ^9^ ^]^ UT‐B facilitates highly efficient transportation of urea.^[^ ^10^ ^]^ Artificially re‐engineered membranes with UT‐B may be a feasible approach to promote urea transportation in bladder tumor cells. Therefore, small interfering RNA targeting CPS1 (siCPS1) co‐delivered with urease may synergistically increase the intracellular deposit of ammonia to a level of cellular toxicity against cancerous cells.

Intravesical instillation is the standard‐of‐care treatment for bladder cancer.^[^ ^11^ ^]^ Bladder tumors can be directly connected to the outside of the body through the urethra, which provides favorable conditions for the development of innovative drugs for the treatment of bladder cancer through intravesical instillation.^[^ ^12^ ^]^ To enhance intravesical drug retention, we sought to construct an instilling nanoparticle‐hydrogel supersystem for BC treatment. To cascade boost the deposit of intracellular ammonia, a biomimetic fusogenic liposomalized nanoporter (FLNP) that was decorated with UT‐B was first synthesized with protonated chitosan oligosaccharide (COS) for bladder tumor‐targeted co‐delivery of urease and siCPS1. A mussel‐inspired hydrogel featured with dual functions of bio‐adhesion and injectability was then fabricated as a reservoir for intravesical immobilization of FLNPs. After intravesical instillation, the adhesive hydrogel adhered to the wet bladder wall. Bld‐1 is a specific targeting peptide for bladder cancer cells, and its affinity for bladder cancer cells is far higher than that for other cells.^[^ ^13^, ^14^ ^]^ The FLNPs liberated from the gel tracked and actively targeted the bladder tumor cells via the Bld‐1 peptide. Subsequently, in a membrane fusion manner, UT‐B was immobilized in cancerous cell membranes. The unshelled nanoporter (NP) then disintegrated, and the cargo, including urease and siCPS1, were released into the cytosol. We found that FLNP‐mediated UT‐B‐cell membrane immobilization dramatically enhanced urea transport into tumor cells, and co‐delivery of urease and siCPS1 significantly boosted the intracellular accumulation of ammonia to an eradicating level against bladder cancer cells (Figure 1f). The multipotencies of the instilling nanoparticle‐hydrogel system were explored by modulation of the metabolic cascade of intracellular ammonia to combat BC in multiple orthotopic bladder tumor models.

## 2 Results and Discussion

**Figure 2:**
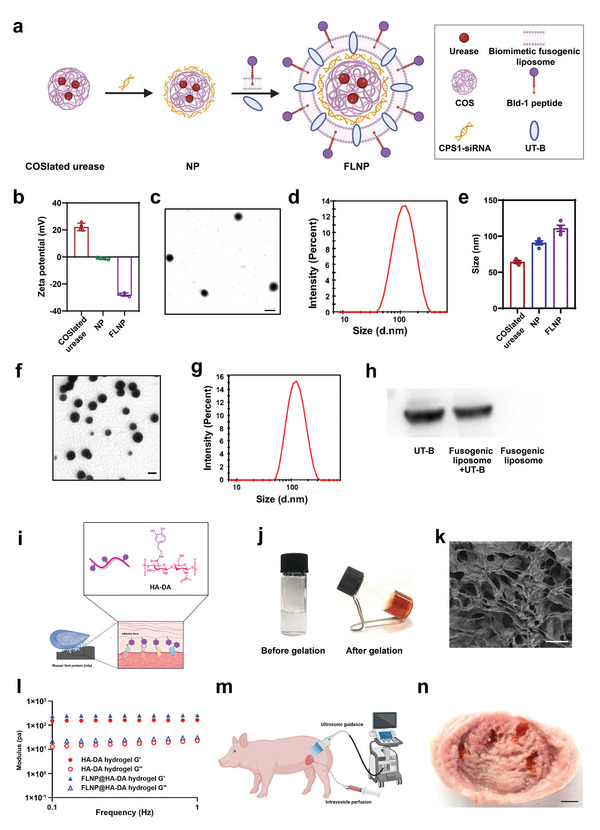
Synthesis procedure and characterization of the intravesical instillation superstructure. a) Schematic illustration showing FLNPs preparation. b) Zeta potential of each formulation (*n* = 4). c) TEM images of NPs. Scale bar indicates 100 nm. d) Dynamic light scattering (DLS) analysis of NPs. e) Particle size distributions of each formulation (*n* = 4). f) TEM images of FLNPs. Scale bar, 100 nm. g) DLS analysis of FLNPs. h) Western blot analysis of UT‐B in different formulations. i) Schematic illustration of the bionic principle of HA‐DA. j) Images showing the gelator solution before gelation (left) and the hydrogel after gelation (right). k) SEM (scale bar, 20 µm) images of the hydrogel. l) Rheology traces of the HA‐DA hydrogels. m) Schematic diagram of ultrasound‐guided intravesical perfusion of the hydrogel. n) The image of bladder dissection showing that the hydrogel adheres to the bladder wall at 1 h after bladder irrigation, scale bar: 1 cm.

COS is an inexpensive and ideal cationic biopolymer material for drug delivery due to its biocompatibility, noncytotoxicity and biodegradability.^[^ ^15^ ^]^ It has been classified by the FDA as generally recognized as safe for humans.^[^ ^16^ ^]^ Inspired by this, we used the preprotonated COS to assemble with urease and form an NP for efficient protein delivery (**Figure** **2**a). To construct the NP, we first prepared COS‐loaded (COSlated) urease by mixing COS (0.2 g mL^−1^) solutions with urease, resulting in NPs with an average size of 64.34 nm, and a uniform shape (Figure S1a,b, Supporting Information). The prepared COSlated urease is positively charged, with a zeta potential of 22.21 mV (Figure 2b). The COSlated urease was then used to condense the negatively charged siCPS1 at different weight ratios and examined on a 2% agarose gel. As shown in Figure S2, Supporting Information, the COSlated urease completely condensed the siRNA at a weight ratio of 8:1, and this result was confirmed by the zeta potential result in Figure 2b, with an average diameter of 90.78 nm (Figure 2c–e). Additionally, we further investigated the changing of urease activity before and after being stirred with COS, and found that there was no significant changing before and after urease being stirred with COS (Figure S3, Supporting Information). We then coated the NPs with a biomimetic fusogenic liposome (BFL) for urea transport by reconstituting UT‐B into the liposome, while the Bld‐1 peptide‐decorated lipid membrane greatly improved the targeting capacity of the NP. Transmission electron micrographs revealed that FLNPs had a spherical vesicular structure with a diameter of ≈111.08 nm (Figure 2f,g), followed by conflation with the fusogenic liposome. Through urease ELISA assay and siRNA assay, we further confirmed the co‐loading of urease and CPS1 siRNA in the FLNP. As shown in Figure S4a,b, Supporting Information, the ELISA and gel assay showed that there were urease and CPS1 siRNA loading in FLNP. According to the drug loading formula, we calculated the drug loading of urease and siRNA in FLNP is 6.94% ± 0.39% and 1.29% ± 0.06% respectively. The presence of UT‐B on the BFL membrane was verified by western blotting and fluorescent antibody detection (Figure 2h and Figure S4c, Supporting Information).

Mytilus is a marine organism that can adhere to wet rock walls. The functionalization of biopolymers containing catechol in bionic mussels has been used to improve the biocompatibility and enhance the adhesion of 3D hydrogels.^[^ ^17^ ^]^ The proteins that play an adhesive role in mussels include repeated catecholic amino acids, lysine residues, and 3,4‐dihydroxy‐L‐phenylalanine (L‐DOPA, DA).^[^ ^18^ ^]^ Hyaluronic acid‐DA (HA‐DA) hydrogel can efficiently adhere to the surface of tissues. This ability mainly depends on the high affinity between the DA group in HA‐DA hydrogel and the diversity nuclei (e.g., amines, thiol, and imidazole), so that the DA group can bind to the peptides and proteins on the surface of tissues (Figure 2i). As shown in Figure S5, Supporting Information, there is a catechol proton‐specific peak at ≈7 ppm in the nuclear magnetic resonance (NMR) assay result, which demonstrates the conjugation of dopamine to HA. As shown in Figure 2j, the color of HA‐DA pregel solution changes from transparent color to brown immediately during gelation due to the oxidative conversion of the catechol moiety into their corresponding *o*‐quinones. We further investigated the modulus changing during the process of hydrogel formation from the liquid phase to the solid phase. As shown in Figure S6, Supporting Information, the cross‐linkers were added after a 3.5 min conditioning step, and the gel points (*G*′ = *G*″) were rapidly reached. As shown in Figure 2k, the HA‐DA hydrogels exhibited a homogeneous porous structure. Afterward, dynamic mechanical analysis was used for determined the HA‐DA hydrogel (2%) rheological properties after gelation. We quantified the modulus of hydrogel in a frequency range from 0.1 to 1 Hz, and found that the storage modulus (*G*′) was constantly higher than the loss modulus (*G*″), demonstrating the stability and viscoelasticity of the HA‐DA hydrogel (Figure 2l). To form the FLNP@HA‐DA hydrogel, we loaded 5 mg mL^−1^ FLNP in the hydrogel. We also tested the FLNP‐hydrogel structure and release behavior. The SEM result clearly show the liposomes loaded on hydrogel (Figure S7a, Supporting Information), and the release experiment indicated that the FLNP sustained release from the hydrogel up to 24 h (Figure S7b, Supporting Information). We further explored the injectability of the hydrogel. As shown in Figure S8a, Supporting Information, the hydrogel passed through an 18G syringe needle smoothly and forming various designed shapes. Furthermore, we detected the content of FLNP in the hydrogel before and after injection by using the method of Dil‐labeled FLNP, and found that the injection had no significant effect on the content of FLNP in the hydrogel (Figure S8b, Supporting Information). To verify the adhesion of the hydrogel to the bladder wall of large animals, we used a pig model. Under the guidance of ultrasound, hydrogel was injected into the bladder of a pig. After 1 h, the bladder was dissected out of the body and cut open; photograph showed the status of the hydrogel in the bladder wall (Figure 2m,n). Additionally, we further investigated the adhesion time of the hydrogel to the bladder wall. As shown in Figure S9, Supporting Information, at 24 h after perfusion, the hydrogel adhered to the bladder wall decreased compared with that at 1 h. And at 48 h, the hydrogel adhered to the bladder wall was significantly reduced to almost completely degraded. This result indicated that the hydrogel can continuously adhere to the bladder wall for at least 24–48 h.

**Figure 3:**
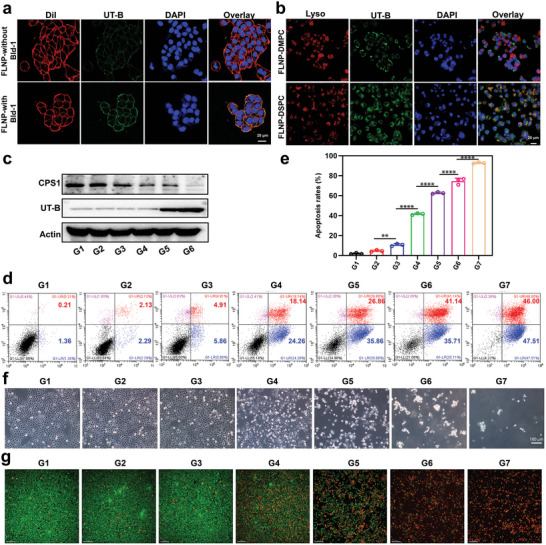
FLNP mediated high levels of UT‐B on the cell membrane and CPS1 inhibition of bladder cancer cells. a) Cellular uptake of FLNP with Bld‐1 or FLNP without Bld‐1 by bladder cancer cells as measured through confocal microscopy. Images from left to right show cell membrane (red), UT‐B (green), DAPI‐stained cell nuclei (blue) and merged images. Scale bar indicates 20 µm. b) Images showing the cell components of bladder cancer cells with FLNP‐DMPC and FLNP‐DSPC treatment. The red signal indicates lysosome, green signal indicates UT‐B, and blue signal indicates nuclei. Scale bar represents 20 µm. c) Western blot showing CPS1 and UT‐B protein expression in bladder cancer cells after different treatments for 24 h. G1: control; G2: COSlated urease; G3: NP; G4: NP‐laden fusogenic liposome; G5: NP‐laden UT‐B reconstituted fusogenic liposome; G6: FLNP. d,e) Apoptosis analysis of bladder cancer cells after 24 h of different treatments in culture medium supplemented with FBS and urea (100 mm) using cytometry (*n* = 3 independent experiments). f) Images of bladder cancer cells after 24 h of different treatments in culture medium supplemented with FBS and urea (100 mm). Scale bar indicates 100 µm. g) Fluorescence images of bladder cancer cells in live/dead staining experiments after 24 h of different treatments in culture medium supplemented with FBS and urea (100 mm). Scale bars indicate 150 µm. d–g) G1: control; G2: COS; G3: COSlated urease; G4: NP; G5: NP‐laden fusogenic liposome; G6: NP‐laden UT‐B reconstituted fusogenic liposome; G7: FLNP. Data are presented as mean ± s.d. **p < 0.01; ****p < 0.0001 by one‐way ANOVA test.

In order to verify the therapeutic effect of our designed FLNP on bladder cancer, we first evaluated the targeting capability of the targeted biomimetic FLNP to bladder cancer cells. To confirm this feature, the cellular uptake of Bld‐1 peptide‐modified FLNPs was explored in bladder cancer (MB49) cells. As a control, FLNPs without the targeting ligand were added to MB49 cells and cultured under similar conditions. After 4 h of incubation with Dil‐labeled cancer cells, the Bld‐1 peptide‐modified FLNPs exhibited a much higher UT‐B fluorescence compared to the slight fluorescence intensity of the FLNPs without Bld‐1 peptide group. Moreover, the green signal from UT‐B in the FLNP group gradually merged with the red circle, indicating that FLNPs could fuse with the MB49 cell membrane (**Figure** **3**a). For the FLNP without Bld‐1 group, although the FLNPs quickly adhered to the cell membrane, fusion was too rare to be observed (Figure 3a), which further confirmed the capability of the active targeting of the Bld‐1 peptide in bladder cancer cells. Furthermore, the different cellular uptake induced by diverse formulations was quantitatively investigated by flow cytometry analysis. The cellular uptake of Bld‐1 peptide‐modified FLNPs was increased compared with that of FLNPs without Bld‐1 (Figure S10, Supporting Information), indicating that the Bld‐1 peptide significantly improved the cellular enrichment of FLNPs due to the preserved tumor‐homing ability of the Bld‐1 peptide. We subsequently confirmed the membrane fusion pathway of the FLNP membrane by confocal laser scanning microscopy (CLSM). When we used the lysosome‐specific probe LysoTracker Red and UT‐B fluorescent antibody to label the cells, the results of confocal microscopy showed that UT‐B and lysosomes rarely co‐localized in the cells, indicating that bladder cancer cells did not through endocytosis to uptake FLNP. However, when we replace the DMPC in FLNP with DSPC, we observed endocytosis and colocalization between the lysosomes and the UT‐B, which further confirmed the fusogenic potential of the FLNP membrane with bladder cancer cells (Figure 3b). As shown in Figure S11, Supporting Information, urease and CPS1 siRNA were initially colocalized after the nanoparticles entered the cytoplasm. After 6 h, the colocalization of urease and CPS1 siRNA decreased, and they were distributed in the cytoplasm, indicating that urease and CPS1 siRNA were released into the cytoplasm. To investigate the capability of FLNPs to deliver siCPS1 into tumor cells and silence CPS1 gene expression, we next examined the cellular gene silencing efficiency of FLNPs in vitro. After 24 h of incubation with different formulations, we used western blotting to analyze CPS1 and UT‐B expression in bladder cancer cells. As shown in Figure 3c, FLNP‐delivered siCPS1 dramatically downregulated the levels of CPS1 in MB49 cells at a siCPS1 concentration of 1.0 µg mL^−1^, while CPS1 expression in cancer cells treated with the NPs was still evident. These results further indicate that FLNPs can transfer siRNA into tumor cells and silence the CPS1 gene with satisfactory efficacy. To better enhance the killing effect of ammonia metabolism blockade on tumor cells, we not only interfered with key enzymes of ammonia metabolism, but also increased the content of urea channels on the tumor cell membrane by means of membrane fusion liposome delivery. After the nanoparticles were administered, UT‐B on the membrane fusion liposome fused with the cell membrane of bladder cancer. After fusion, UT‐B was highly expressed on bladder cancer cells (Figure 3c), which provided a channel for the rapid passage of high concentrations of urinary urea to the inside of tumor cells.

As shown in Figure 3d,e, the cells treated with NPs showed ≈42.40% cell apoptosis, and the apoptosis rate of cells treated with NP‐laden UT‐B‐reconstituted fusogenic liposomes increased to 76.85%, while FLNPs induced up to 93.51% cell apoptosis. For the cell viability assay, only moderate anticancer activity was observed in the COS or COSlated urease group, while the NPs exhibited a higher death rate than the COSlated urease, indicating that the siRNA can increase ammonia accumulation, leading to a synergistic therapeutic effect (Figure 3f). In addition, the cells treated with the NP‐laden UT‐B reconstituted fusogenic liposome showed a higher cell proliferation inhibition effect compared with NP‐laden fusogenic liposome (Figure S12a,b, Supporting Information), suggesting that UT‐B reconstituted fusogenic liposome on the NP may increase the urease cellular uptake and subsequently enhance the ammonia concentration. This suggested that the antitumor efficacy was greatly enhanced with the increased intracellular ammonia concentration due to the tumor‐selective targeting of Bld‐1. Moreover, a live/dead viability/cytotoxicity assay for fluorescence microscopy analysis was performed to reveal the cytotoxicity mechanism. As shown in Figure 3g, FLNPs induced significant death in bladder cancer cells with urea supplements (100 mm). Additionally, we investigated the toxicity of FLNP to normal cells. Compared with bladder cancer cells, FLNPs were significantly less toxic to the normal urothelial cell line SV‐HUC‐1 (Figure S13, Supporting Information).

**Figure 4:**
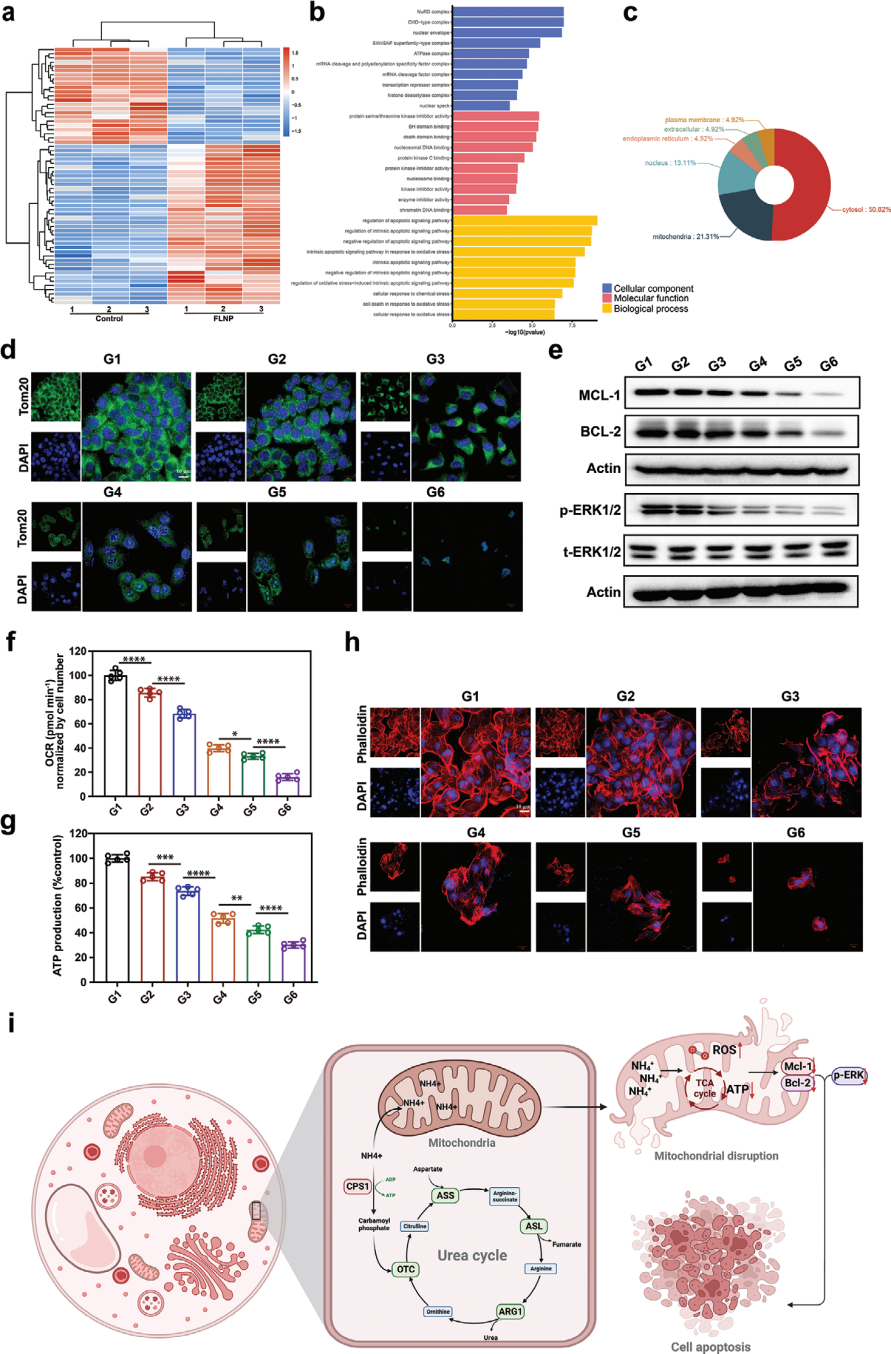
FLNPs induced tumor cell apoptosis mainly by mitochondrial destruction. a) Hierarchical clustering analysis based on protein expression of bladder cancer cells treated with FLNPs compared with the PBS treatment group after 24 h (*n* = 3). b) Significantly enriched GO term histogram (FLNP treated vs PBS) (top 10). c) Subcellular localization analysis of differentially expressed proteins. d) Confocal images of staining for the mitochondrial structural protein Tom‐20 after different treatments for 24 h (scale bar, 10 µm). e) MCL‐1, BCL‐2, and p‐ERK1/2 protein expression levels in bladder cancer cells after different treatments for 24 h. f) FLNPs inhibit cellular oxygen consumption in bladder cancer cells with urea supplements (100 mm). After a 24 h incubation with FLNPs or control agents, the cellular OCR was measured by a Seahorse analyzer (mean ± s.d., *n* = 5). g) Relative cellular ATP level after 24 h of different treatments in culture medium supplemented with FBS and urea (100 mm) (mean ± s.d., *n* = 5). h) Confocal analysis of cellular F‐actin stained by phalloidin after 24 h of different treatments in culture medium supplemented with FBS and urea (100 mm). The scale bar indicates 10 µm. i) Schematic illustration of the FLNPs destroying mitochondria that lead to bladder cancer cell apoptosis. d–h) G1: control; G2: COSlated urease; G3: NP; G4: NP‐laden fusogenic liposome; G5: NP‐laden UT‐B reconstituted fusogenic liposome; G6: FLNP. Data are presented as mean ± s.d. *p < 0.05; **p < 0.01; ***p < 0.001; ****p < 0.0001 for one‐way ANOVA test.

To investigate the underlying mechanism of the FLNPs effects on bladder cancer progression, we first determined ammonia accumulation at the whole protein level. An examination was performed on proteins differentially expressed in tumor cells treated with FLNPs compared to those treated with PBS. A total of 38 upregulated proteins and 23 downregulated proteins were filtered out by setting the t test *p*‐value < 0.05 and the cut‐off value of log2 (fold change) >1 (**Figure** **4**a). Enrichment of the differentially expressed proteins was investigated by pathway database analysis and ranked by *p*‐value. GO enrichment analysis indicated that most of these metabolites were associated with apoptosis process (Figure 4b). Through pathway enrichment analysis, we found that after FLNP treatment, certain signaling pathways including mitochondrial membrane permeability, apoptotic chromosome condensation and apoptotic signaling pathways, were significantly changed. As shown in Figure 4c, the results of organelle localization analysis of the altered proteins showed that the altered proteins were mainly concentrated in the mitochondria and cytoplasm after treatment with FLNP. Mitochondria are not only the main organelles supplying energy to the cell, but also the key organelles involved in ammonia metabolism in the cell, playing an important role in the regulation of ammonia metabolism in the cell.^[^ ^19^, ^20^ ^]^ To further verify the proteomic effects of FLNPs on mitochondria, we further examined the intracellular changes in mitochondria by CLSM. JC‐1 staining is a classical method for displaying mitochondrial membrane potential.^[^ ^21^ ^]^ As shown in Figure S14, Supporting Information, mitochondrial membrane potential was significantly decreased (cell staining changes from red to green) in bladder cancer cells after being treated with FLNPs, indicating remarkable mitochondrial damage by ammonia. Tom‐20 is located at the outer membrane of mitochondria to facilitate the introduction of proteins across the outer membrane of mitochondria, and is a key protein in the executive function of mitochondria.^[^ ^22^ ^]^ Our results showed that FLNPs could significantly reduce Tom‐20 expression in bladder cancer cells, indicating that nanoparticles can strongly damage the mitochondria of bladder cancer cells (Figure 4d and Figure S15, Supporting Information). Moreover, FLNPs also decreased the expression of MCL‐1 and BCL‐2 in tumor cells (Figure 4e), which are involved in mitochondria‐mediated apoptosis. The ERK1/2 signaling pathway controls diverse primary cellular processes such as cell survival, proliferation, fate determination, and stress responses.^[^ ^23^ ^]^ Aberrant ERK1/2 signaling underlies a wide range of important diseases in humans, including aging and cancer.^[^ ^24^ ^]^ In the present study, we revealed that p‐ERK1/2 protein expression was significantly decreased by treatment with FLNPs (Figure 4e). Taken together, our results revealed that FLNPs could induce the apoptosis of bladder cancer cells by disturbing the mitochondria/ERK1/2 signaling pathway.

We further examined the oxygen consumption rate (OCR) and ATP levels in bladder cancer cells after different treatments. As shown in Figure 4f,g, the relative percentages of OCR and ATP production of bladder cancer cells in the FLNP‐treated group were the lowest compared to other groups, which is consistent with previous findings. Moreover, ATP production in MB49 cells also fell sharply in the FLNP group (Figure 4g), demonstrating an efficient mitochondrial respiratory depression effect. The cytoskeleton carries out multiple functions including assisting cell movement and cell morphology changing, connecting the cell biochemically and physically to the external environment and spatially organize the contents of the cell.^[^ ^25^ ^]^ We used phalloidin to stain the cytoskeleton in bladder cancer cells and found that the cytoskeleton was significantly damaged and disordered after FLNP treatment (Figure 4h). Mitochondria produce up to ninety percent of ROS in living cells,^[^ ^26^ ^]^ and our data showed that the bladder cancer cells in the FLNP‐treated group generated more ROS than those in the other groups (Figure S16, Supporting Information). Together, these results indicated that FLNP could efficiently induce mitochondrial damage and inhibit the OXPHOS metabolic pathway, resulting in decreased cellular oxygen consumption and ATP production of bladder cancer cell (Figure 4i).

**Figure 5:**
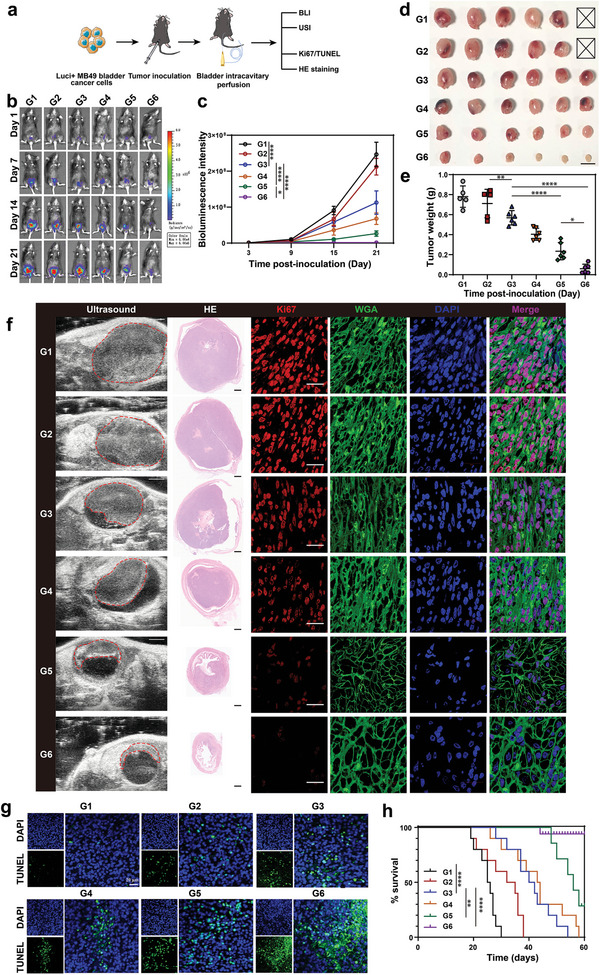
Antitumor efficacy of the intravesical instillation nanoparticle‐hydrogel system in an orthotopic bladder cancer mouse model. a) Diagram showing the experimental procedure in vivo. b) Bioluminescent images and c) quantified diagram of tumor‐bearing mice with different treatment (*n* = 5). Statistical significance was calculated using a Two‐way ANOVA. d) Images of bladder tumors harvested from mice with different treatment (*n* = 5 in G1 and G2; *n* = 6 in G3–G6). Scale bar indicates 1 cm. e) Statistical diagram of tumor weight (mean ± s.d., *n* = 5 in G1 and G2; *n* = 6 in G3–G6). f) Tumor images from left to right showing the ultrasonography, H&E‐staining slides and immunofluorescence which red signal indicates Ki67, green signal indicates WGA and blue signal indicates DAPI. Scale bars in ultrasonography represent 2 mm, H&E staining represent 500 µm, and immunofluorescence images represent 10 µm. g) Immunofluorescence images showing TUNEL staining of bladder tumors. Scale bar represents 20 µm. h) Kaplan–Meier survival analysis of the bladder cancer mouse model treated with different formulations (*n* = 10). log‐rank (Mantel–Cox) test. b–h) G1: control; G2: COSlated urease with hydrogel; G3: NP with hydrogel; G4: NP‐laden fusogenic liposome with hydrogel; G5: NP‐laden UT‐B reconstituted fusogenic liposome with hydrogel; G6: FLNP with hydrogel. Data are presented as mean ± s.d. *p < 0.05; **p < 0.01; ****p < 0.0001 for two‐way ANOVA test, one‐way ANOVA test and Kaplan–Meier survival analysis.

Based on the encouraging results that FLNP induced bladder cancer cell apoptosis by ammonia accumulation, we further explored the therapeutic effect of hydrogel‐loaded FLNP on bladder cancer after irrigating into the bladder using the orthotopic MB49 bladder cancer mouse model (**Figure** **5**a). As shown in Figure 5b, we used the IVIS lumina system to monitor tumor growth in different treatment groups of bladder cancer‐bearing mice. Quantitative analysis of bioluminescence signals showed that the tumor‐bearing mice in the FLNP hydrogel treatment group had the lowest tumor fluorescence intensity compared with the other five groups, indicating the strongest inhibitory effect on bladder cancer (Figure 5c and Figure S17, Supporting Information). Although the average BLIS was slightly lower than that of the PBS control group, no significant difference was obtained in the COSlated urease group (Figure 5c). Moreover, the average BLIS for the NP‐laden UT‐B reconstituted fusogenic liposome group and the FLNP group were 25.7 and 1.2 times larger than the original tumors, respectively. After FLNP‐hydrogel treatment, the tumors also showed the lowest weight and size, consistent with the ultrasound imaging results in vivo (Figure 5d–f). In addition, the results of Ki67 staining of tumor sections showed that the Ki67‐positive cells of mouse tumors in the FLNP hydrogel treatment group were lowest, indicating that the proliferation of bladder cancer was dramatically inhibited (Figure 5f). In terminal deoxynucleotidyl‐transferase‐mediated dUTP nick‐end labeling (TUNEL)‐stained bladder tumor sections, the most severe apoptosis was detected in the FLNP hydrogel treatment group (Figure 5g). Therefore, benefiting from the remarkable effect of shrinking tumor, the survival time of bladder cancer bearing mice treated with FLNP hydrogel was prominently prolonged (Figure 5h). Together, these results demonstrated that FLNP‐mediated ammonium accumulation exhibited superior antitumor activity. In addition, there were no significant pathological changes in the main organs of the mice with different drugs treatment compared with the mice from control group (Figure S18a, Supporting Information). It is worth mentioning that the peripheral blood biochemical indicators also showed that no significant differences were found in the levels of glutamic pyruvate transaminase (ALT), aspartate transaminase (AST), glucose (GLU), UREA, creatinine (CREA) and alkaline phosphatase (ALP) in the serum of mice after diverse treatments (Figure S18b, Supporting Information). Both of these results preliminarily revealed the good biocompatibility of the FLNPs and validated the biosafety of the nanoparticle‐hydrogel treatment system.

**Figure 6:**
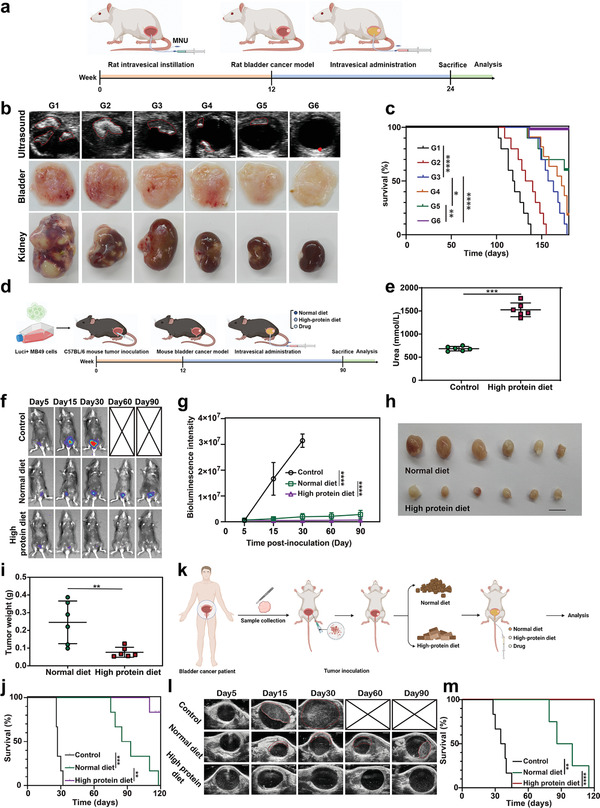
A high protein diet enhances the antitumor effect of the intravesical instillation nanoparticle‐hydrogel system in bladder cancer mouse models. a) Schematic illustration of the experimental design in the rat bladder cancer model induced by MNU. b) Ultrasound images and macroscopic images of excised bladder‐ and kidney‐bearing tumors. c) Kaplan–Meier survival analysis of tumor‐bearing rats in different groups (*n* = 10). log‐rank (Mantel–Cox) test. d) Schematic illustration of the experimental design in the MB49 tumor‐bearing mouse model treated with the intravesical instillation superstructure and different dietary protein levels. e) Urea concentration in the urine of mice fed a normal or high protein diet (*n* = 6). Student's *t*‐test. f) Bioluminescent images and g) quantified diagram of tumor‐bearing mice with different treatment (*n* = 5), including control group, FLNP hydrogel with normal diet group and FLNP hydrogel with high protein diet group. Statistical significance was calculated via two‐way ANOVA. h) Images of bladder tumors harvested from mice with different treatment (*n* = 6). Scale bar indicates 1 cm. i) Statistical diagram of tumor weight from mice with different treatment (mean ± s.d., *n* = 6). Student's *t*‐test. j) Kaplan–Meier survival analysis of bladder cancer mice model treated with intravesically instilled FLNP‐hydrogel in normal or high protein diet (*n* = 6). log‐rank (Mantel–Cox) test. k) Diagram showing the experimental procedure of PDX mouse model. l) Ultrasound images of orthotopic bladder tumors in a PDX mouse model treated with intravesically instilled FLNP‐hydrogel and a normal or high protein diet. Scale bars, 2 mm. m) Kaplan–Meier survival analysis of orthotopic bladder tumors in a PDX mouse model treated with the intravesically instilled nanoparticle‐hydrogel system and a normal or high protein diet (*n* = 6). log‐rank (Mantel–Cox) test. b,c) G1: control; G2: COSlated urease with hydrogel; G3: NP with hydrogel; G4: NP‐laden fusogenic liposome with hydrogel; G5: NP‐laden UT‐B reconstituted fusogenic liposome with hydrogel; G6: FLNP with hydrogel. (c,e,g,i,j,m) Data are presented as mean ± s.d. *p < 0.05; **p < 0.01; ***p < 0.001; ****p < 0.0001 for Kaplan–Meier survival analyse, Student's *t*‐test and two‐way ANOVA test.

Next, we further investigated whether intravesical irrigation of FLNP could exert an effective antitumor effect in female Sprague–Dawley rat models of the chemical carcinogen *N*‐methyl‐*N*‐nitrosourea (MNU) induced orthotopic bladder cancer (**Figure** **6**a). We used ultrasound imaging of the rat bladder to monitor the development of tumors in the bladder of rats in different treatment groups. As shown in figure 6b, it summarized the effects of different treatment drugs on the orthotopic rat bladder tumors. During a period of 12 weeks, tumors in all groups developed gradually, and up to 24 weeks, it was clearly shown in the ultrasound imaging results that nearly half of the bladder inner space was occupied by diffuse bladder tumors. Comparatively, the tumors significantly shrunk in the other two groups, including the NP‐laden UT‐B reconstituted fusogenic liposome and FLNP groups. COSlated urease and NPs exhibited relatively weak inhibitory effect on bladder cancer. Excitingly, ultrasound imaging showed that the tumors in the FLNP treatment group were almost completely eliminated. In order to more intuitively verify the effect of FLNP on bladder tumors, we dissected the bladder from the sacrificed rats and cut the bladder open to better observe the tumors on the inner wall. As shown in Figure 6b, large numbers of tumors can be clearly seen on the inner wall of bladder from the control group rats, while there could hardly observed tumors on the inner wall of the bladder from the FLNP administration group, which further confirmed the therapeutic effect of FLNP on bladder cancer. Importantly, we also found severe renal metastasis in the MNU induced orthotopic rat bladder cancer model treated with PBS, whereas no metastatic tumors in kidney after treating with FLNPs (Figure 6b). As shown in Figure 6c, the survival time of FLNP treated rats with bladder cancer was significantly longer than that of other treatment groups. The above results indicated that the FLNP treatment showed superiority anti‐tumor and anti‐metastasis performance.

Urea is a major nitrogenous waste product of protein metabolism.^[^ ^27^ ^]^ The above results demonstrate that delivery of urea to tumor cells through UT‐B while blocking the decomposition of urea and increasing the accumulation of ammonium can kill tumors effectively. Hence, we hypothesis that high‐protein diet increases the urea concentration in urine, which inspires us to explore whether the continuous high urea stimulation could increase the antitumor efficacy of FLNP (Figure 6d). In tumor‐bearing mice fed with high‐protein diet, urea was highly concentrated in urine up to 1525.21 mmol L^−1^ (Figure 6e). While mice that fed with regular diet and water, urinary urea concentration was ≈684.38 mmol L^−1^. The urea concentration in urine of mice on a high‐protein diet was ≈twofold higher than that of mice on a normal diet. Compared with the rapid tumor growth curve of the normal diet group, group fed the high‐protein diet showed a relatively smooth growth curve. As shown in Figure 6f–i and Figure S19, Supporting Information, the high‐protein group led to significant tumor regression. The image and weight results of tumor also show that high protein diet can significantly enhance the therapeutic effect of FLNP on bladder cancer (Figure 6h,i). Consequently, the mice treated with FLNPs in the high‐protein diet group exhibited significantly prolonged survival, indicating that the high protein diet resulted in improved antitumor effects of FLNPs, largely due to the enhanced urea concentration and intracellular ammonia accumulation induced by synergistic anticancer efficacy of urease and siCPS1 (Figure 6j).

To further test the effect of a high protein diet on the antitumor efficacy of FLNPs, we established an aggressive orthotopic bladder tumor model of patient‐derived xenografts (PDXs) that closely mimicked the tumors from cancer patients (Figure 6k). PDX bladder cancer model in NOG mice were established by using surgical specimens obtained from patient with bladder cancer undergoing radical cystectomy. The mice were then divided into three groups, followed by various treatments. Encouragingly, treatment with FLNPs and a high protein diet produced the greatest inhibition of tumor growth; the treatment had significantly higher inhibitory ratios than those from the PDX model receiving a normal diet (Figure 6l and Figure S20a,b, Supporting Information). We further monitored the effects of different treatments on the long‐term survival time of bladder cancer‐bearing mice. The FLNP‐treated tumor‐bearing mice in the high protein diet group survived longer than those in the normal diet group (Figure 6m). These data confirm that a high protein diet contributes toward inhibiting tumor growth in PDX models, which is favorable for maximizing the therapeutic efficacies of synergistic ammonium metabolism treatment. To further validate the translational potential of our study, protein powder supplementation was performed preoperatively for bladder cancer patients undergoing radical cystectomy. Patient urine samples were collected before and after a high‐protein diet. As shown in Figure S21, Supporting Information, the urea concentration of urine in the high protein diet group was much higher than that in the normal diet group. These results suggest that instilling nanoparticle‐hydrogels in combination with a high protein diet holds promise for the clinical treatment of bladder cancer.

## 3 Conclusions

In summary, we reported an intravesical instillation nanoparticle‐hydrogel hybrid system for cascade metabolic modulation of intracellular ammonia to combat bladder cancer. We found that the FLNP‐mediated UT‐B immobilization dramatically induced urea transportation into tumor cells, and co‐delivery of urease and siCPS1 significantly boosted ammonia accumulation in tumors inducing cell apoptosis. Treatment with the hybrid system exhibited superior antitumor effects in an orthotopic bladder tumor mouse model and a PDX model, respectively. Combined with high‐protein diet increased the production of urinary urea, lead to an augmented intracellular deposition of ammonia in BC cells, and ultimately an enhanced tumor inhibition. Together, our work established that harnessing urinary urea tumor‐tropistic transportation and its ammonia transformation could induce tumor apoptosis and may be a practical strategy for eradication of BC with TP53 mutation.

## 4 Experimental Section

### Materials

COS, hydrochloride, and HA were purchased from Shanghai Macklin Biochemical Co., Ltd.; DOTAP and DSPE‐PEG‐NHS were obtained from Shanghai Aiweite Pharmaceutical Technology Co., Ltd.; the 2′,7′‐dichlorofluorescein diacetate (DCFH‐DA), propidium iodide (PI), calcein AM, JC‐1, and ATP assay kits were purchased from Solarbio Life Sciences. Annexin V/PI Apoptosis Detection Kit was purchased from BD Bioscience. The Bld‐1 peptide was purchased from GenScript Biotechnology Co., Ltd.; siRNA targeting CPS1 mRNA was synthesized by TSingke Biotechnology Co., Ltd.; and DAPI, Ki67 and TUNEL kits were obtained from ThermoFisher. Dil, LysoTracker and WGA lectin were provided by Dalian Meilun Biotech Co., Ltd and Gene Tex. The UT‐B, CPS1, MCL‐1, BCL‐2, p‐ERK1/2, and t‐ERK1/2 antibodies for western blot analysis were obtained from Abcam or Cell Signaling Technology.

### Synthesis of COSlated Urease

0.1 mg mL^−1^ urease was added to protonated COS solution (0.2 g mL^−1^) and stirred at room temperature for 30 min. Afterward, glutaraldehyde was added to the abovementioned mixed solution as the cross­linker. Finally, the COSlated urease was purified and concentrated by ultracentrifuge.

### Synthesis of DSPE‐PEG‐NHS‐Bld‐1

In brief, the conjugate was prepared by dissolving 5 mg of Bld‐1 and 20 mg of DSPE‐PEG‐NHS in DMF at room temperature, and triethylamine was added to adjust the pH to 7–8 with slow stirring. After stirring in the dark for 3 days, the obtained mixture was dialyzed at 3500 Da cut off molecular weight in deionized water for 48 h. Finally, the obtained product was lyophilized and kept at −20 °C.

### Construction of the Nanoporter

For the urease and siRNA‐coloaded NP, the siRNA was condensed with the COSlated urease at various weight ratios (1:1, 2:1, 4:1, 8:1, and 16:1) at room temperature for 30 min to form urease and siRNA‐coloaded NPs. The formation of the NP was confirmed using a gel retardation assay on a 2% agarose gel.

### Liposomal Coating

DMPC, DSPE‐PEG‐Bld‐1, and DOTAP were mixed at a molar ratio of 65:3:17 to prepare the fusogenic liposome coatings. The lipid films were synthetized by evaporating the solvent and hydrating with payload‐NP solution. Then the NP‐hydrated lipid was kept heating to 40 °C for 10 min with stirring. The collected mixture was physically extruded through a 200 nm polycarbonate membrane for 20 times in total.

To construct the FLNP, UT‐B (isolated and purified from red blood cell membranes) was reconstituted onto the fusogenic liposome as previously reported.^[^ ^28^ ^]^ Briefly, 1 mg UT‐B was mixed with FLNPs in 1 mL HEPES buffer solution. Then, the suspension was mixed to homogeneity and quickly frozen in liquid nitrogen for 5 min, followed with subsequently sonicating in a bath sonicator for 30 s. Finally, the suspension was centrifuged at 2000 rpm, and washed three times to remove the free UT‐B.

DLS (Zetasizer ZS90, Malvern Instruments) was used to measure nanoparticle size and zeta potential, and JEM‐1200 EX II electron microscope was used to visualize the structural morphology. In short, 5 µL samples were dropped onto the TEM grid and dried off excess solvent after 1 min. The experiment was replicated four times and averaged.

### Co‐loading of Urease and Carbamoyl Phosphate Synthetase 1 in Fusogenic Liposomalized Nanoporter Assay

FLNP was dissociated using 1% Triton‐X100 in PBS and the mixture was placed in a shaker at 300 rpm min^−1^ for 2 h to dissociate the FLNP and release of urease and siRNA. After being centrifuged at 12 000 rpm at 4 °C for 10 min, the liposome‐free supernatant was collected. The urease was detected in the supernatant by using ELISA (Abcam, cat: ab34806). And the siRNA was detected by gel assay and Nanodrop One C Microvolume UV–vis spectrophotometer.

### Synthesis and Gelation of Hyaluronic Acid‐DA Conjugate

Briefly, 0.5 g HA was dissolved in 0.5 m MES buffer to form 1% HA solution (w/v). Then, 0.25 g EDC and 0.15 g NHS were added to HA solution as prepared above, and the mixture was stirred for 30 min at pH 5.0. Next, DOPA·HCl was added to the mixture, followed by stirring overnight at room temperature at pH 5.0. Unreacted DOPA·HCl was removed through dialysis against 1× PBS three times. The final product of the HA‐DA conjugate was confirmed using NMR. For hydrogel preparation, HA‐DA conjugate dissolved in PBS, NaIO4 at a 1:1 molar ratio to DOPA were mixed thoroughly to form the 2% HA‐DA hydrogel (w/v). Rheometer (Anton Paar MCR302) was used to detect the mechanical properties of HA‐DA hydrogel. The value of dynamic moduli (storage modulus *G*′ and loss modulus *G*″) was obtained.

### Patients

The Ethics Committee of the Qilu Hospital of Shandong University approved all research processes and specimens’ collection in this study (KYLL‐2021‐267). All the patients involved in this study provided informed consent. Urine from bladder cancer patients was collected on admission. Then, the patients were given a high‐protein diet as required for 2 days. After that, the urine from patients was collected again. Fresh bladder tumor samples were obtained through surgery for further experiments.

### Animals

6‐ to 8‐week‐old C57BL/6 mice and female Sprague–Dawley (SD) rats were obtained from the Laboratory Animal Center of Shandong University. 6‐week‐old female Cg‐Prkdcscid Il2rg^tm1Sug/JicCrl^ (NOG) mice were obtained from Vital River Laboratory Animal Technology. All animal experiments were approved by the ethics committee of the Qilu Hospital of Shandong University (KYLL‐2020‐264).

### Cell Culture

The human bladder cancer cell line T24 was obtained from American Tissue Culture Collection (ATCC) and cultured in McCoy's 5A with 10% fetal bovine serum (FBS). The mouse bladder cancer cell line MB49 were purchased from Millipore and maintained in RPMI‐1640 media supplemented with 10% FBS.

### In Vitro Cell Proliferation Assay

To detect the cell proliferation, cells were seeded in 96‐well plates at a density of 3 × 10^3^ cells per well. After different treatments for 48 h, the plates were fixed with precooled trichloroacetic acid, and then stained with 0.4% sulforhodamine B in 1% acetic acid. The absorbance was determined at a wavelength of 560 nm in 10 mm Tris‐based solution.

### Apoptosis Assay

Annexin V/PI apoptosis detection kit was used to determine the cell apoptosis. In brief, cells were cultured in 6‐well plates and treated differently. After washing, the single‐cell suspension was collected and incubated with Annexin V and PI successively according to the instruction. Then, the apoptotic rates were analyzed by flow cytometry.

### Western Blot Analysis

After different treatment, cells were lysed in RIPA buffer supplemented with protease inhibitors on ice, and centrifuged at 12 000 × *g* to remove the debris. The supernatant was mixed with SDS buffer and boiled for 10 min. Samples were resolved in polyacrylamide gel electrophoresis, then incubated with indicated primary and secondary antibodies. The blots were visualized using detection reagents.

### Oxygen Consumption Measurement

Seahorse XFe96 Flux Analyzer was used to measure the OCR according to standard protocols. Briefly, cells were seeded into XFp Seahorse plates and treated differently for 24 h. Before measurement, the cells were washed with PBS and changed to seahorse base medium free of phenol red and bicarbonate followed by equilibration for 30 min in a 37 °C incubator without CO_2_. Then, the value of cellular OCR was obtained.

### ATP Assay

For the detection of intracellular ATP levels, the bioluminescent ATP assay kit (FLASC, Sigma) was used following the standard protocol. Briefly, cells were seeded in 6‐well plates along with the indicated treatment. For the measurement of cellular ATP levels, the cells were collected and resuspended in lysis reagent, and the ATP extracts were mixed with luciferase reagent. Then, the luminescence signals were measured by an IVIS Spectrum imaging system.

### Confocal Laser Scanning Microscope Analysis

For fluorescence imaging, bladder cancer cells were grown on glass coverslips, treated differently, and then fixed and stained with different fluorescent dyes as needed.

### Microfilament Structure Staining

For visualization of microfilament structures, after being washed with PBS, the cells were incubated with phalloidin in PBS for 2 h followed by DAPI staining for 1 min at room temperature. After three washes, the cells were mounted and analyzed using a confocal microscope for microfilament and nuclear morphology using a 63× objective.

### In Vitro Mitochondrial Membrane Potential Staining

The cyanine dye JC‐1 (5, 5′, 6, 6′‐tetrachloro‐1, 1′, 3, 3′‐tetraethylbenzimi‐ dazolylcarbocyanine iodide) is a fluorescent probe for mitochondrial behavior, which can change from normal green fluorescent dye to red fluorescent aggregate when response to higher mitochondria membrane potential. In this study, a confocal microscope was used to display the alternation of mitochondria. Briefly, the cells were cocultured with JC‐1 at 37 °C for 15 min followed by DAPI staining at room temperature and then washed for imaging on a confocal microscope.

### Urea Transporter‐B Location/Colocalization Assay

To examine the UT‐B location on bladder cancer cells, cells were stained with UT‐B antibody. Cell membranes were stained with Dil, and nuclei were stained with DAPI. Following rinsing with PBS, the colocalization of the cell membrane and UT‐B was evaluated under a microscope.

### Lysosome Staining

Cells were incubated with LysoTracker red and UT‐B antibody (labeled by a fluorescent secondary antibody) for 30 min. After staining the nucleus with DAPI, images were captured with a confocal microscope.

### Ki67 Staining

For fluorescence imaging, the tissue sections were deparaffinized by xylene, dehydrated in ethanol, and boiling for antigen retrieval. After blocking, the sections were incubated with anti‐Ki67 antibody and plasma membrane probe WGA at 4 °C, followed with DAPI staining for nuclei. The confocal microscopy was used to capture the images.

### Terminal Deoxynucleotidyl‐Transferase‐Mediated dUTP Nick‐End Labeling Staining

One Step TUNEL Apoptosis Assay Kit was used for DNA fragmentation and cell apoptosis detection. In brief, the samples were treated with TdT enzyme and stained, followed with DAPI staining for nuclei. Fluorescent images were detected by visualization with a confocal microscope.

### Orthotopic Bladder Cancer Mouse Model

The bladder cancer mouse model was established by intravesical injection of bladder cancer cells. Briefly, the anesthetized mice were inoculated with luciferin‐expressing MB49 cells into the bladder wall using insulin syringes.

### Chemically Induced Bladder Cancer Rat Model

The most common preclinical animal model of bladder cancer uses a nitrosamine compound, MNU, which causes high‐grade and invasive tumors of the urinary bladder. Briefly, under anesthesia, rat bladders were catheterized with disposable venous catheters, and then the MNU solution was irrigated into bladder through a sterile syringe. The rats were administered MNU every 2 weeks.

### Patient‐Derived Xenograft Mouse Model

A PDX bladder cancer mouse model was established using NOG mice. The surgical specimens from bladder cancer patients’ tumors were collected and divided into small pieces and then transplanted subcutaneously into anesthetized NOG mice. Once the tumors were grown in F0 mice, the xenografts were collected and further transplanted into another mouse for expansion. In general, F2‐ or F3‐ generation PDX tumors were used for further study.

### Tumor‐Bearing Animal Model Treatment

Several days after model establishment, the animals were grouped according to the tumor burden and treated regularly. For bladder perfusion, the anesthetized animals were catheterized and perfused with different formulations of the nanoparticle‐hydrogel hybrid system. Mice were sacrificed when the primary tumors reached a threshold, and the bladders were harvested for following experiments.

### Bioluminescence Live Imaging

The tumor burdens were measured using bioluminescence imaging with an IVIS Spectrum imaging system (PerkinElmer). After intraperitoneally injected with D‐luciferin, mice were anesthetized and detected for luminescence signal. The Living Image Software was used for the images analyzed.

### Ultrasound Imaging

Bladder tumor growth was monitored, and the antitumor effect of FLNPs was assessed using ultrasound imaging. Under anesthesia, the bladders were catheterized and distended with PBS using venous catheters and sterile syringes for imaging. Each animal was placed in the supine position on the platform, and the Vevo LAZR high‐resolution ultrasound system was used to acquire the images.

### Hematoxylin and Eosin Staining

At the animal experimental end point, the bladder tumors and main organs were collected for further analysis. After fixation, the tissues were embedded in paraffin, sliced into sections and histologically examined following hematoxylin and eosin staining.

### Immunohistochemical Staining

The samples were sectioned, deparaffinized, dehydrated and boiled in citrate buffer. Then immunohistochemical staining was performed using human polyclonal antibodies against CPS1. All images were acquired using a VS120 Olympus microscope (Olympus).

### Urea Nitrogen Detection

Urea is hydrolyzed by urease to produce ammonium ions and carbon dioxide, and ammonium ions react with phenol chromogenic agents to form blue substances in alkaline environments. The amount of the blue substance was directly proportional to the content of urea, which was determined by colorimetry at a wavelength of 640 nm. The concentration of urea nitrogen in urine was detected by a urea assay kit following the manufacturer's instructions.

### Blood Biochemistry Analysis

≈1 mL of blood from each mouse was collected. The upper sera were collected by centrifuging at 3000 rpm for subsequent biochemical analysis. The serum levels of urea nitrogen (UREA), creatinine (CREA), aspartate aminotransferase (AST), alanine aminotransferase (ALT), alkaline phosphatase (ALP) and glucose (GLU) were evaluated using an automatic chemistry analyzer (BS‐240VET).

### Statistics Analysis

Data analyses in this study were carried out with GraphPad Prism 8.0. The results are presented as the mean ± standard deviation (s.d.) and based on experiments performed at least in triplicate, *n* = 3–50 depending on the condition being presented. Difference between two groups were analyzed using Student's *t*‐test. One‐way ANOVA test was used to analyze differences among multiple groups. Two‐way ANOVA test was used to compare differences between two or more groups with different time points. Kaplan–Meier survival analyses were performed with the log‐rank test and used for survival data of every group. Figure legends specify the statistical strategies and data presentation. *p* < 0.05 was considered statistically significant (*p < 0.05; **p < 0.01; ***p < 0.001; ****p < 0.0001).

## Conflict of Interest

X.J., W.J. and C.C. are inventors on patent application submitted by Shandong University that covers the following content: Metabolic Modulation of Intracellular Ammonia Eradicates Bladder Carcinoma in a Murine Model of Patient‐Derived Xenograft. The other authors declare that they have no other competing interests.

## Author Contributions

W.J. and C.C. contributed equally to this work. X.J., B.S., W.J., and C.C. conceived the project and designed the experiments. W.J., C.C., G.W., M.H., S.C., M.A., X.J., C.S., P.S., and Z.Y. performed the experiments. W.J., C.C., G.W., M.H., B.S., and X.J. analyzed and interpreted the data in this study. W.J., C.C., B.S., and X.J. wrote the manuscript draft.

## Supporting information

Supporting Information

## Data Availability

The data that support the findings of this study are available from the corresponding author upon reasonable request.
