# Supplementary material for: Metabolic Modulation of Intracellular Ammonia via Intravesical Instillation of Nanoporter‐Encased Hydrogel Eradicates Bladder Carcinoma
Source: Adv Sci (Weinh). 2023 Feb 12;10(12):2206893. doi: 10.1002/advs.202206893 (PMC10131795; doi:10.1002/advs.202206893)
Supplement: Supplementary file 1 — Supporting Information [file ADVS-10-2206893-s001.pdf]

Supporting Information

Metabolic Modulation of Intracellular Ammonia via Intravesical Instillation of Nanoporter-Encased Hydrogel Eradicates Bladder Carcinoma

Weiqiang Jing[#], Chen Chen[#], Ganyu Wang, Maosen Han, Shouzhen Chen, Xin Jiang, Chongdeng Shi, Peng Sun, Zhenmei Yang, Benkang Shi[*], Xinyi Jiang[*]

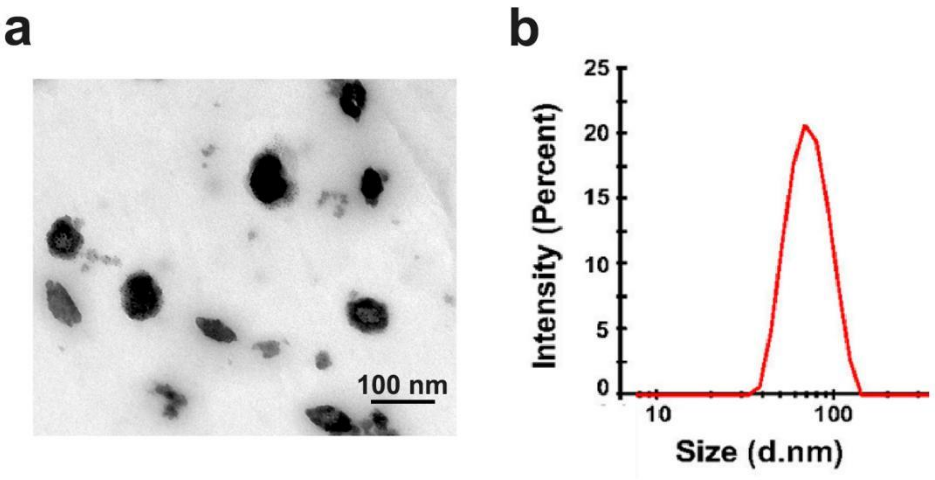

Figure S1. Material characterization of COSlated urease. (**a**) TEM images of COSlated urease. Scale bar, 100 nm. (**b**) Dynamic light scattering analysis (DLS) of COSlated urease.

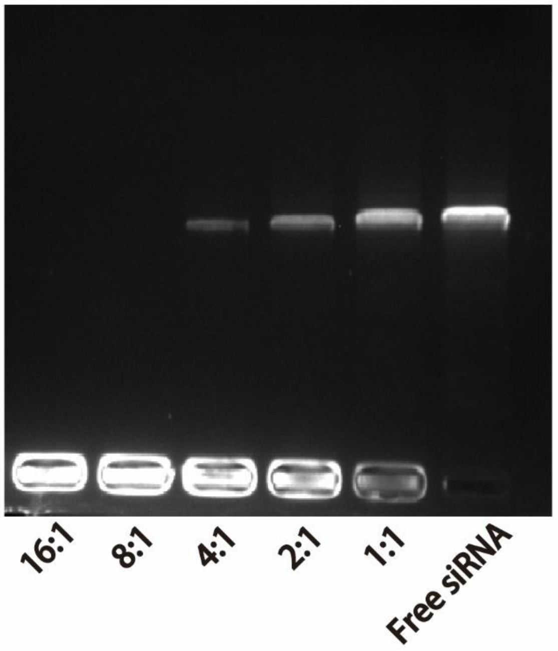

Figure S2. Gel retardation assay of the siRNA-laden nano-porter.

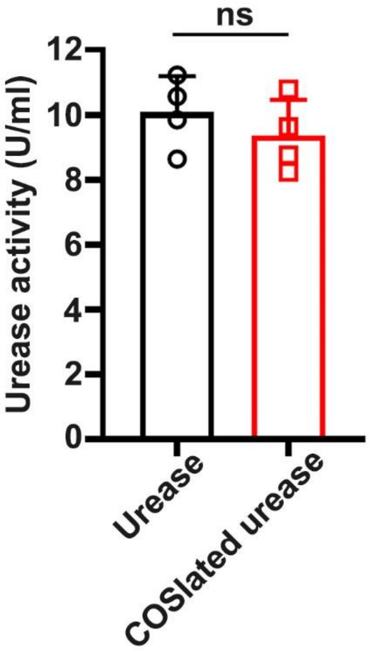

Figure S3. Urease activity before and after being stirred with COS (mean ± s.d., n=4). Ns. no significance.

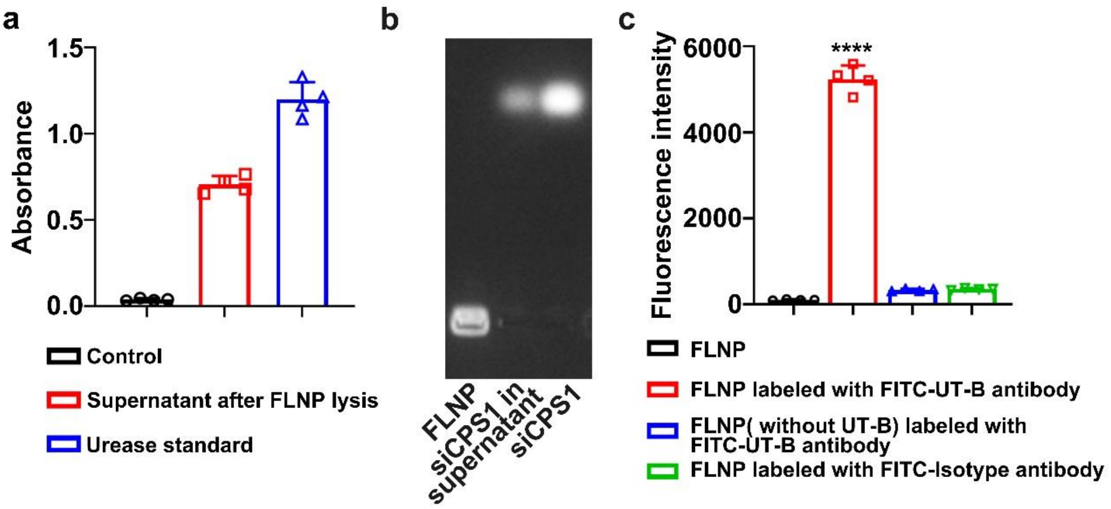

Figure S4. Materials characterization of FLNP loading. (**a**) Urease ELISA assay of control (PBS), supernatant after FLNP lysis, and Urease standard (mean ± s.d., n=4). (**b**) Gel retardation assay of FLNP, supernatant after FLNP lysis, and CPS1 siRNA. (**c**) The fluorescence intensity of different samples (mean ± s.d., n=4). ****P<0.0001.

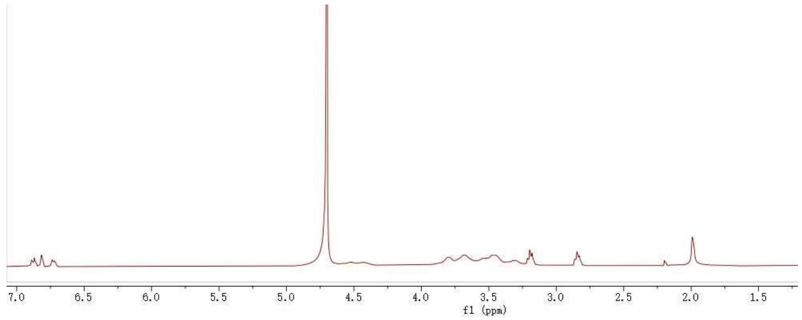

Figure S5. ^{1}H-NMR spectra of the HA-DA.

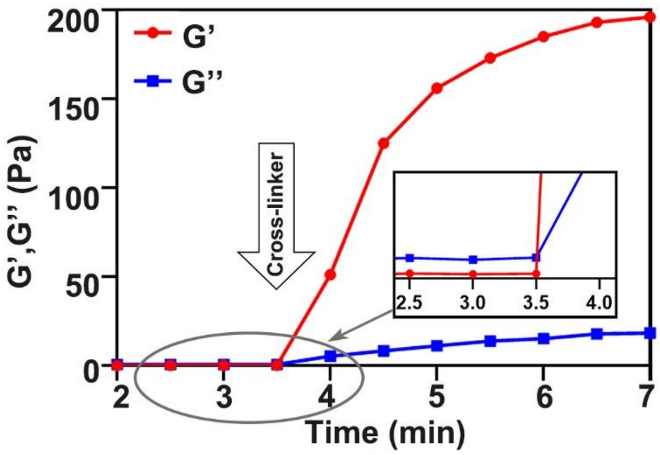

Figure S6. Rheological characterization of the initial crosslinking kinetics of HA-DA by cross-linker ($NaIO_4$) in oscillation mode with a constant frequency of 1 Hz. The cross-linker was added at 3.5 min after the start of the measurement.

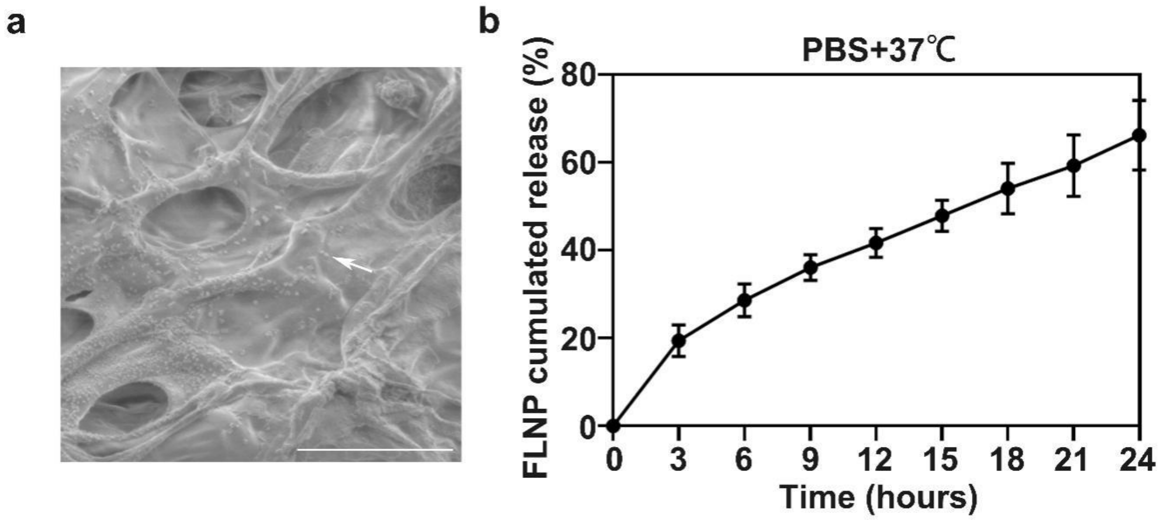

Figure S7. Characterization of FLNP-hydrogel. (**a**) SEM images of the FLNP-hydrogel. Arrow indicates liposome. Scale bar = 20 μm. (**b**) Drug release behavior of FLNP from the hydrogel (mean ± s.d., n=3).

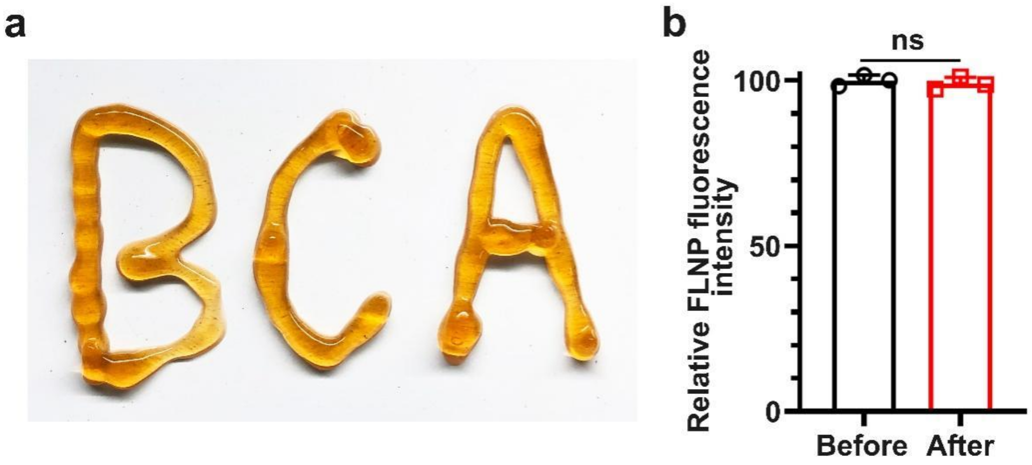

Figure S8. The injectability of FLNP hydrogel. (**a**) The hydrogel was injected through a syringe needle and formed into various shapes. (**b**) Relative FLNP fluorescence intensity in the hydrogel before and after injection (mean ± s.d., n=3). Ns. no significance.

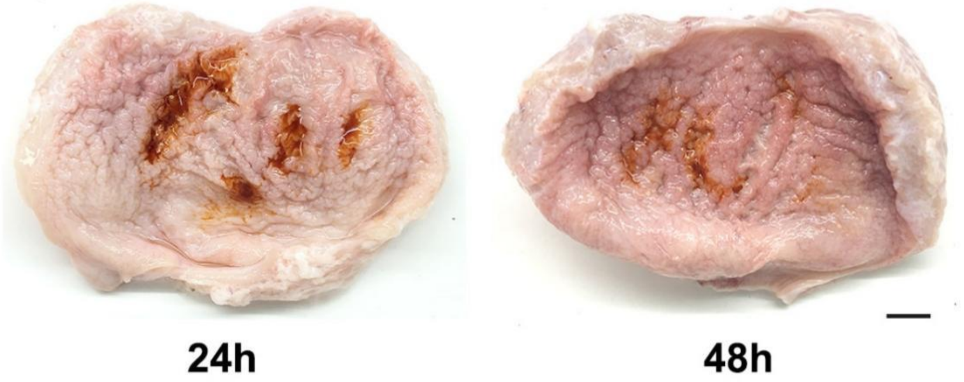

Figure S9. Image of bladder dissection showing that the hydrogel adheres to the bladder wall at 24 and 48 hours after bladder irrigation; scale bar: 1 cm.

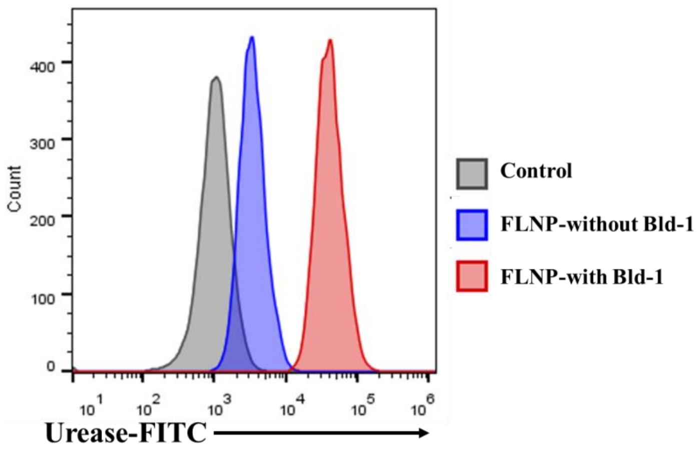

Figure S10. Cellular uptake analysis in bladder cancer cells after treatment with different formulations using flow cytometry.

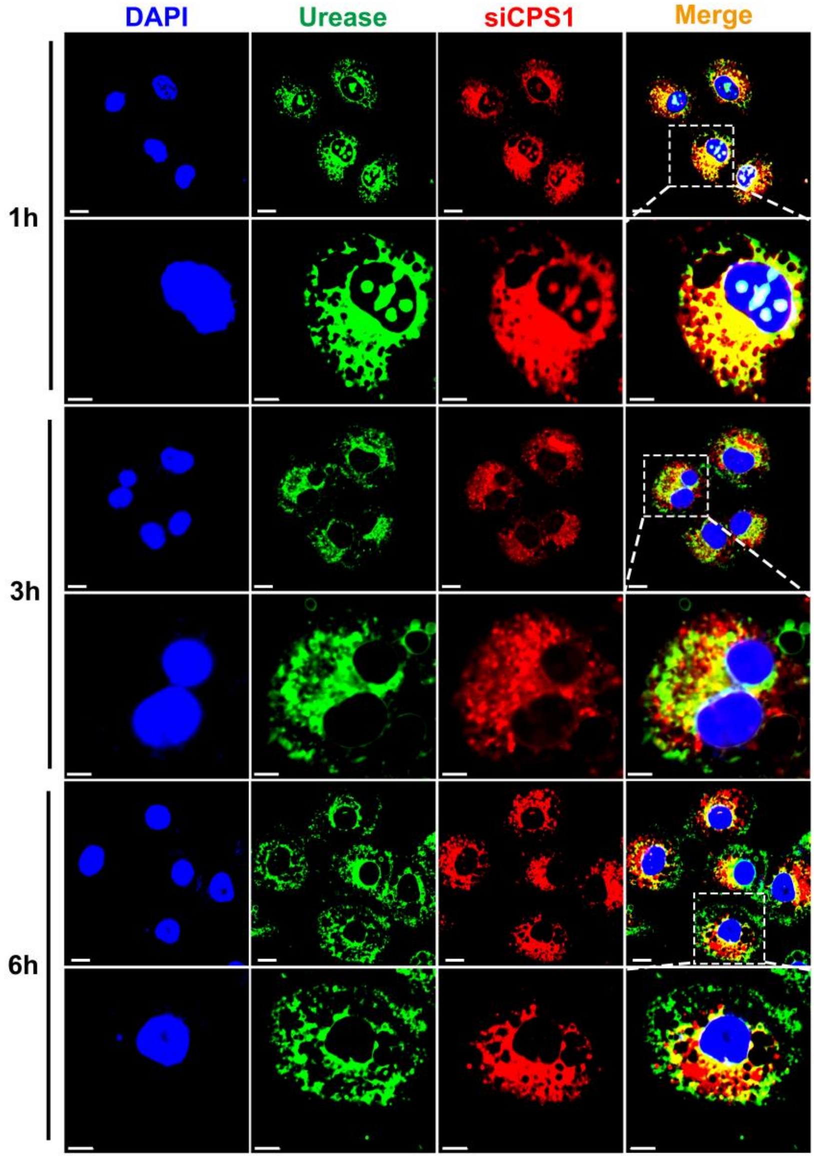

Figure S11. Confocal images of subcellular compartments of bladder cancer cells after coincubation with FLNP. Images from left to right show DAPI-stained cell nuclei (blue), urease (green), siCPS1 (red), and merged images. Scale bars in low magnification images represent 10 μm, and in high magnification images represent 5 μm.

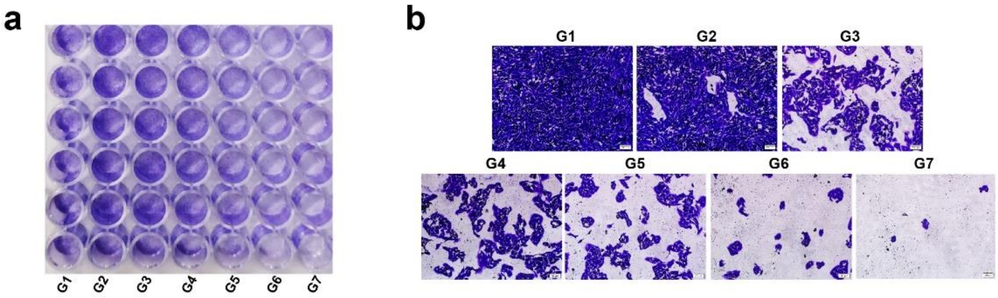

Figure S12. FLNP inhibited bladder cancer cell proliferation. (**a**) Cell viability assay used crystal violet staining of bladder cancer cells after 48 h of different treatments in culture medium supplemented FBS and urea (100 mM). (**b**) Images of bladder cancer cells after different treatments. Scale bars indicate 50 μm. G1: Control; G2: COS; G3: COSlated urease; G4: NP; G5: NP-laden fusogenic liposome; G6: NP-laden UT-B reconstituted fusogenic liposome; G7: FLNP.

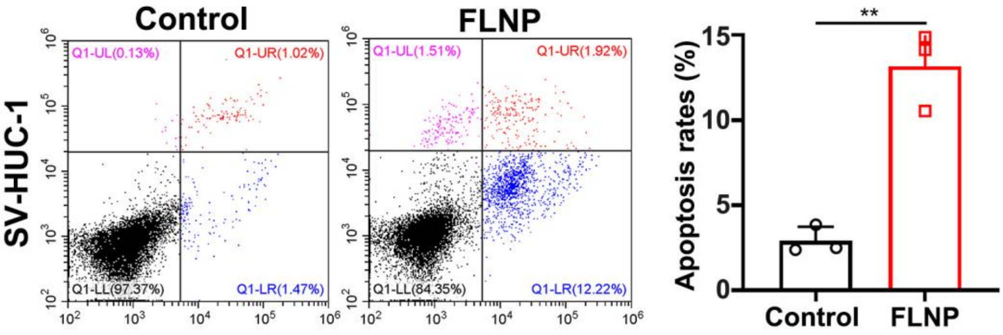

Figure S13. Apoptosis analysis of SV-HUC-1 cells after 24 hours of different treatments in culture medium supplemented with FBS and urea (100 mM) using cytometry (mean ± s.d., n=3). **P<0.01.

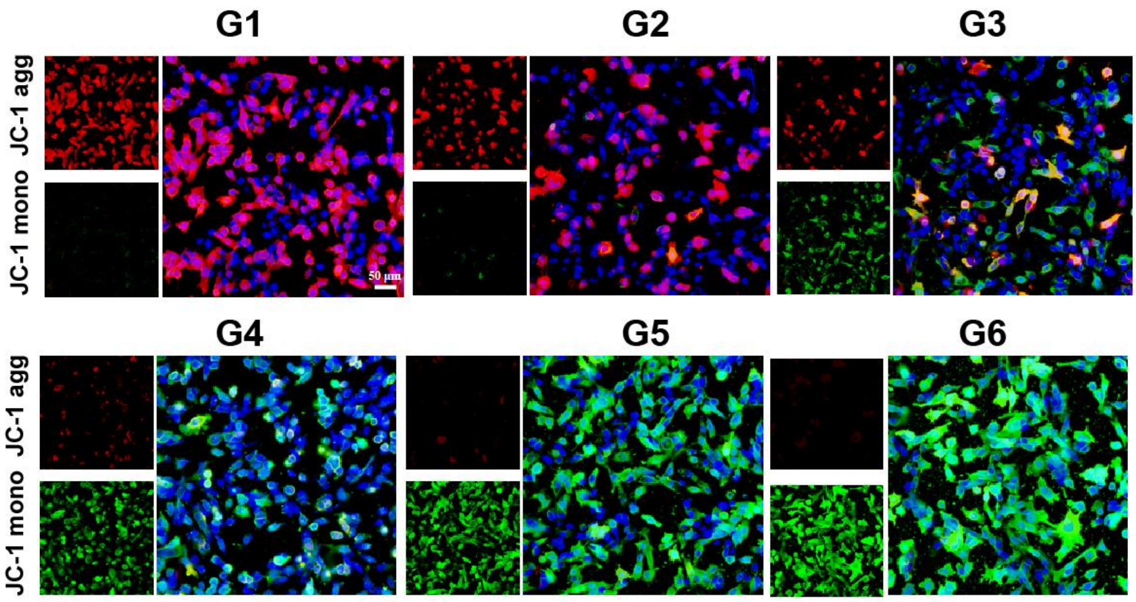

Figure S14. Mitochondrial membrane potential of MB49 cells after 6 h of incubation with FLNP with urea supplements (100 mM) or other control agents, indicated by JC-1 staining (10 μg/ml). Red fluorescence that is emitted by aggregated JC-1 characterizes a high membrane potential while green fluorescence emitted by JC-1 monomer characterizes low membrane potential (scale bar, 50 μm). G1: Control; G2: COSlated urease; G3: NP; G4: NP-laden fusogenic liposome; G5: NP-laden UT-B reconstituted fusogenic liposome; G6: FLNP.

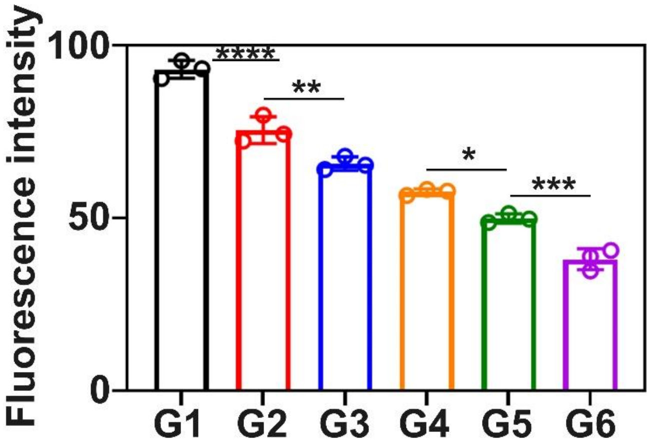

Figure S15. Tom-20 fluorescence intensity of individual cells from treatment groups (mean ± s.d., n=3). G1: Control; G2: COSlated urease; G3: NP; G4: NP-laden fusogenic liposome; G5: NP-laden UT-B reconstituted fusogenic liposome; G6: FLNP. *P<0.05; **P<0.01; ***P<0.001; ****P<0.0001.

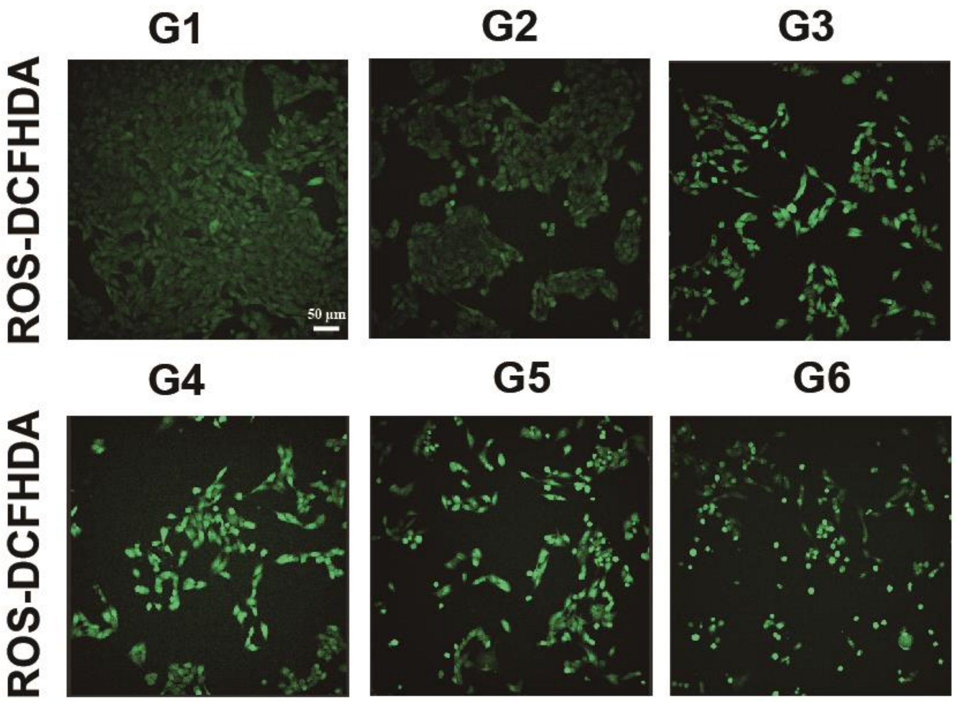

Figure S16. Confocal analysis of ROS detected by DCFHDA after 24 h of different treatments in culture medium supplemented FBS and urea (100 mM) (scale bar, 50 μm). G1: Control; G2: COSlated urease; G3: NP; G4: NP-laden fusogenic liposome; G5: NP-laden UT-B reconstituted fusogenic liposome; G6: FLNP.

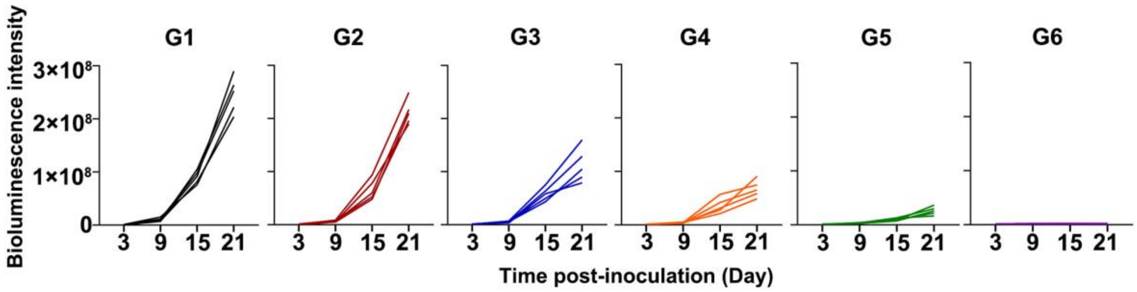

Figure S17. In vivo bioluminescence quantified signal intensity (n=5). G1: Control; G2: COSlated urease with hydrogel; G3: NP with hydrogel; G4: NP-laden fusogenic liposome with hydrogel; G5: NP-laden UT-B reconstituted fusogenic liposome with hydrogel; G6: FLNP with hydrogel.

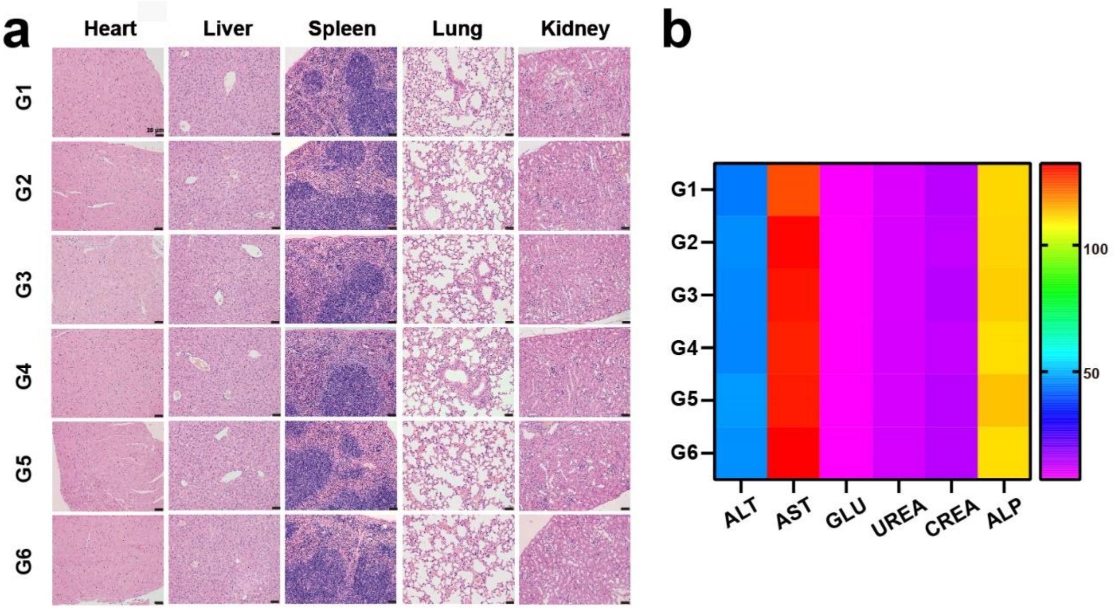

Figure S18. Safety evaluation of drugs in mice. (**a**) The H&E staining of major organs that were isolated from the mice treated with each formulation at day 21 post tumor implantation (scale bars, 100 μm). (**b**) Heat map representing the serum biochemical index of the mice treated with different group (n=4). G1: Control; G2: COSlated urease with hydrogel; G3: NP with hydrogel; G4: NP-laden fusogenic liposome with hydrogel; G5: NP-laden UT-B reconstituted fusogenic liposome with hydrogel; G6: FLNP with hydrogel.

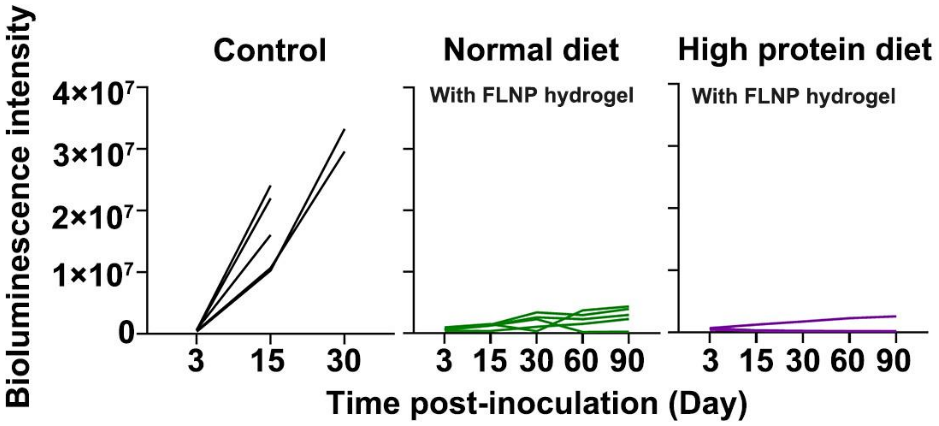

Figure S19. In vivo bioluminescence quantified signal intensity (n=5).

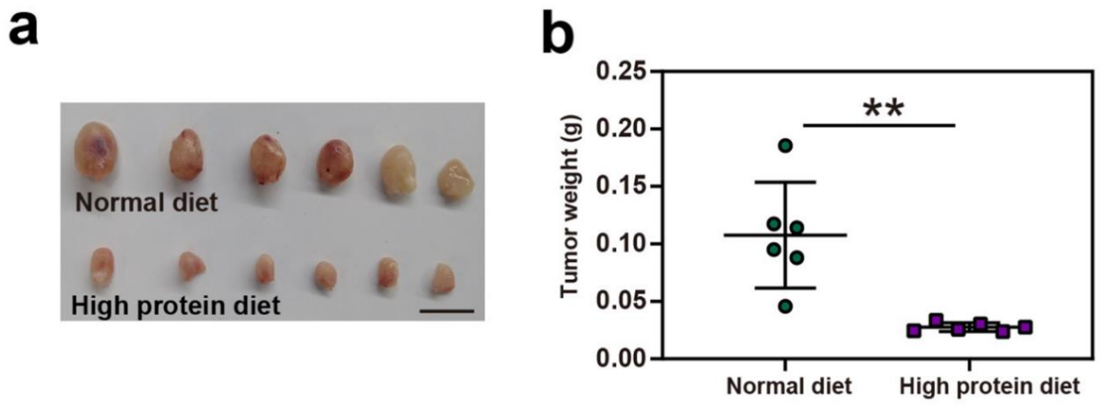

Figure S20. High protein diet enhanced tumor inhibition induced by the nanoparticle-hydrogel system. (**a**) Macroscopic image of excised bladder tumors. Scale bar indicates 1 cm. (**b**) Tumor weight after different treatment (mean ± s.d., n=6), Student's t-Test, **P<0.01.

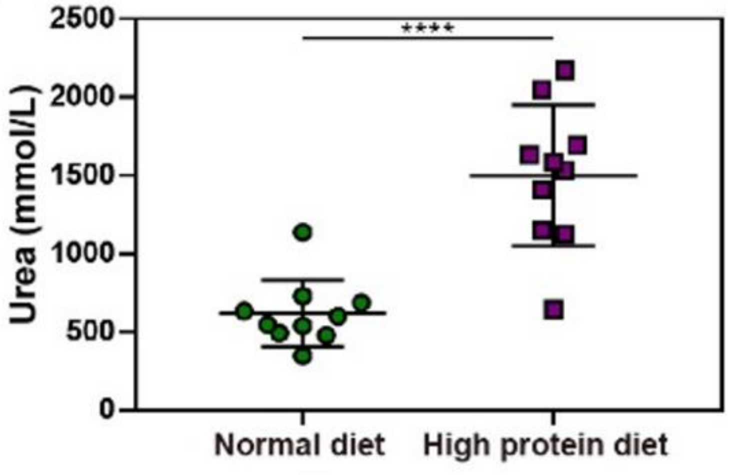

Figure S21. Urea concentration in urine from bladder cancer patients with normal or high protein diet. (mean ± s.d., n=10), Student's t-Test, ****P<0.0001.
